# Integrative immune transcriptomic classification improves patient selection for precision immunotherapy in advanced gastro-oesophageal adenocarcinoma

**DOI:** 10.1038/s41416-022-02005-z

**Published:** 2022-10-17

**Authors:** Manuel Cabeza-Segura, Valentina Gambardella, Francisco Gimeno-Valiente, Juan Antonio Carbonell-Asins, Lorena Alarcón-Molero, Arturo González-Vilanova, Sheila Zuñiga-Trejos, Pilar Rentero-Garrido, Rosana Villagrasa, Mireia Gil, Ana Durá, Paula Richart, Noelia Alonso, Marisol Huerta, Susana Roselló, Desamparados Roda, Noelia Tarazona, Carolina Martínez-Ciarpaglini, Josefa Castillo, Andrés Cervantes, Tania Fleitas

**Affiliations:** 1 Department of Medical Oncology, Hospital Clínico Universitario, INCLIVA, Biomedical Research Institute, University of Valencia, Valencia, Spain; 2 CIBERONC, Instituto de Salud Carlos III, Madrid, Spain; 3 Cancer Evolution and Genome Instability Laboratory, UCL Cancer Institute, London, UK; 4 Department of Bioinformatics and Biostatistics. INCLIVA, Biomedical Research Institute, Valencia, Spain; 5 Department of Pathology, INCLIVA, Biomedical Research Institute, University of Valencia, Valencia, Spain; 6 Department of Precision Medicine, INCLIVA, Biomedical Research Institute, Valencia, Spain; 7 Department of Gastroenterology and Hepatology, Hospital Clínico Universitario de Valencia, Valencia, Spain; 8 Department of Medical Oncology, Hospital General Universitario, Valencia, Spain; 9 Department of Gastroenterology and Hepatology, Hospital General Universitario, Valencia, Spain; 10 Department of Medical Oncology, Hospital Universitario y Politécnico La Fe, Valencia, Spain; 11 Department of Gastroenterology and Hepatology, Hospital Universitario y Politécnico La Fe, Valencia, Spain; 12 Department of Biochemistry and Molecular Biology, University of Valencia, Valencia, Spain

**Keywords:** Cancer microenvironment, Gastric cancer

## Abstract

**Background:**

Advanced gastro-oesophageal cancer (GEA) treatment has been improved by the introduction of immune checkpoint inhibitors (CPIs), yet identifying predictive biomarkers remains a priority, particularly in patients with a combined positive score (CPS) < 5, where the benefit is less clear. Our study assesses certain immune microenvironment features related to sensitivity or resistance to CPIs with the aim of implementing a personalised approach across CPS < 5 GEA.

**Design:**

Through integrative transcriptomic and clinicopathological analyses, we studied in both a retrospective and a prospective cohort, the immune tumour microenvironment features. We analysed the cell types composing the immune infiltrate highlighting their functional activity.

**Results:**

This integrative study allowed the identification of four different groups across our patients. Among them, we identified a cluster whose tumours expressed the most gene signatures related to immunomodulatory pathways and immunotherapy response. These tumours presented an enriched immune infiltrate showing high immune function activity that could potentially achieve the best benefit from CPIs. Finally, our findings were proven in an external CPI-exposed population, where the use of our transcriptomic results combined with CPS helped better identify those patients who could benefit from immunotherapy than using CPS alone (*p* = 0.043).

**Conclusions:**

This transcriptomic classification could improve precision immunotherapy for GEA.

## Background

Gastro-oesophageal adenocarcinoma (GEA) represents the fourth leading cause of cancer death worldwide [1]. Despite significant advances in multimodal approaches and efforts to personalise treatment, overall survival for metastatic patients is still poor [2]. In the field of immunotherapy, patients with microsatellite instability (MSI-H) have shown greater response to immune checkpoint inhibitors (CPIs) across different lines [3]. Recently, the combination of platinum-based chemotherapy and immunotherapy as first line strategy has demonstrated a clear improvement in both progression-free survival (PFS) and overall survival (OS) across GEA [4–6]. Furthermore, a randomised trial has also demonstrated the potential role of nivolumab (anti-PD1) as an adjuvant maintenance strategy in improving PFS in patients with resected stage II or III oesophageal or gastro-oesophageal junction cancer with prior neoadjuvant chemoradiotherapy and residual pathological disease [7]. Overall, it was demonstrated that GEA patients presenting a combined positive score (CPS) > 5 benefit the most from CPIs. However, a lower benefit was seen in the CPS ≥ 1 subgroup [6, 8]. There is therefore a pressing need to identify biomarkers predictive of response in patients presenting with a CPS > 1 and <5, as resistance is a frequent event in this group [9, 10]. An important factor in determining resistance to immunotherapy is the complexity of the tumour immune microenvironment, which influences tumour response to therapies. Conventionally, so-called “cold tumours” are characterised by an immune desert or immunosuppressive profile which contribute to immune tolerance and progression; in contrast, the presence of infiltrating immune cells indicates strong immune system activity against tumours [11, 12]. Beyond the ability to determine the presence or lack of immune-infiltrate, therefore, improved understanding the function of immune cells composing the tumour microenvironment, as well as of cell–cell interactions is required to move precision immunotherapy forward. For this reason, there is a growing interest in identifying both immune microenvironment and tumour components that could serve as biomarkers able to predict response to standard chemotherapy and CPIs. In the present study, our aim was to investigate the cellular and functional immune characteristics of the tumour microenvironment, moving to a precision immunotherapy approach. By performing in-depth transcriptomic study, we propose a novel immune classification for advanced GEA patients, which in combination with CPS could potentially improve the prediction of response to CPIs.

## Methods

### Patient characteristics and study design

Eligible patients were selected according to the following inclusion and exclusion criteria: male and female patient aged ≥18 years, chemotherapy naive, metastatic, with histologically confirmed GEA, with a CPS < 5 (Supplementary Fig. 1) and no comorbidities that could potentially impair the analyses. This study was designed with two cohorts: a retrospective one collecting 31 paraffin-embedded samples available for molecular analyses from January 2004 to January 2019, and a second prospectively collected cohort in which fresh frozen tissues from 23 patients were obtained from January 2020 until September 2021. In both cohorts, chromosomal instability (CIN) cases were defined as MSS/EBV-negative gastric adenocarcinomas, and genomic stable (GS) cases were defined as MSS/EBV-negative diffuse-type gastric cancers [13]. Blood samples in the second cohort were collected prior to any oncological treatment. All participants provided written informed consent. The study protocol was approved by our Institutional Review Board. Samples were stored at the Incliva biobank. An external third cohort was used to validate our findings [14]. This study included 45 patients who after progressing to at least a first-line platinum-based regimen were treated with a checkpoint-inhibitor as a second or third line.

### Sample preparation and transcriptomic analyses

To perform the nCounter analysis, RNA was isolated from five 10-µm-thick FFPE slides using the RNeasy FFPE Kit (cat. no. 73504, Qiagen) following the manufacturer’s instructions. Its integrity and concentration were evaluated by RNA ScreenTape (Agilent). Appropriate input was used according to the NanoString protocol, and we conducted the nCounter® PanCancer Immune Profiling panel v1.1 which included 770 genes covering both the adaptive and innate immune response (NanoString Technologies, Inc., Seattle, WA).

To perform RNA-sequencing analysis, RNA from fresh-frozen tissues was extracted by RNeasy micro kit (cat. no. 74004, Qiagen). Qualities of total RNA samples were determined using an RNA ScreenTape (Agilent technologies). Polyadenylated (poly(A)) RNA was purified using the NEBNext Poly(A) messenger RNA (mRNA) Magnetic Isolation Module (E7490L, NEB). First-strand and second-strand cDNA was synthesised following the NEBNext Ultra II RNA Library Prep Kit (E7770). The library quality was assessed using the HSD1000 ScreenTape (Agilent technologies) and quantification was performed using a QuantiFluor dsDNA Kit (Promega) on a Glomax Discovery fluorometer (Promega). Libraries were then pooled and size-selected to adjust the final library molar concentration for sequencing. Finally, paired-end sequencing was performed in a NextSeq 550 platform (Illumina) using v2.5 chemistry to a length of 150×2.

### Pathological analysis and tumour microenvironment evaluation by immunohistochemistry (IHC)

IHC staining was performed on the formalin-fixed paraffin-embedded tissue of 54 GEA using the automated Autostainer Link 48 system (Dako, Glostrup, Denmark). The following antibodies were used: MLH1 (monoclonal mouse, ES05, prediluted, Dako), MSH2 (monoclonal mouse, FE11, prediluted, Dako), MSH6 (monoclonal rabbit, EP49, prediluted, Dako), PMS2 (monoclonal rabbit, EP51, prediluted, Dako), CD3 (polyclonal, rabbit anti-human prediluted, Dako), CD8 (monoclonal mouse, C8/144B, prediluted, Dako), CD163 (monoclonal mouse, diluted 1/100, BioCare Medical), HER2 (4B5, prediluted, Ventana-Roche), FOXP3 (D6O8R, 1/100, Cell signaling), and PD-L1 (monoclonal mouse anti-PD-L1, 22C3, prediluted, Dako) [15, 16]. For MSS/MSI, only complete loss of nuclear staining with positive internal control was considered loss of mismatch repair (MMR) protein expression. For PD-L1, the combined positive score (CPS) was assessed, as previously described [17]. The percentage of expression in neoplastic and inflammatory cells was also recorded independently. For CD163, the number and percentage of positive cells in one hotspot 40× field was assessed. The number and percentage of CD3, CD8 and FOXP3 positive cells were studied in an area of 600 µm by applying the automated artificial intelligence algorithm for “positive cell count” developed by the Qupath software application (https://qupath.github.io). For EBER evaluation, ISH was performed and interpreted as described in our previous work [18]. HER2 was assessed following consensus recommendations for gastro-oesophageal neoplasms [16]. All results were confirmed by two dedicated pathologists.

### Transcriptomic bioinformatic analyses

NanoString gene expression data was log_2_-transformed and normalised using the housekeeping genes as control. Tumour immune infiltrate was assessed by immune cell scores defined by NanoString using nSolver v3.0 analysis software (NanoString Technologies, Inc.). Each functional signature, also predefined by NanoString, was studied by an unsupervised hierarchical clustering heatmap and patients were classified as high function or low function according to the cluster corresponding to each patient. Heatmaps were performed using Heatmap.plus R package.

For RNA-sequencing analyses, raw RNA expression data of the external CPI-exposed cohort (PRJEB25780) were downloaded from Tumour Immune Dysfunction and Exclusion (http://tide.dfci.harvard.edu/). The same procedure was conducted for both this external dataset and our prospective cohort. Raw RNAseq reads were processed with fastp v0.20.1 [19] to remove low quality bases and adaptor sequences. Transcript abundance was calculated with kallisto 0.46.1 [20]. Gene count matrices were built with tximport v1.22 [21]. ComBat-seq [22] was used on the raw counts in order to correct for batch effects. The corrected counts per gene were then normalised by the variance stabilising transformation (VST) method included in the DESeq2 v1.34.0 package [23]. To identify the presence of infiltration of immune cells, the Immune score from the ESTIMATE package was calculated [24] using the TPM values obtained with ComBat-Seq. Differential expression analysis was conducted with the DESeq2 v1.34.0 [23] package, using an adjusted *p* value cutoff of 0.05 and an absolute log2 fold change over 1. Functional enrichment analysis on differentially expressed genes was done with clusterProfiler v4.2.2 [25], using a hypergeometric (one sided) test with a Benjamini–Hochberg adjusted *p* value of 0.05. Unsupervised clustering heatmaps were performed using Heatmap.plus R package.

### Blood cytokine profile by Luminex Technology

Blood was drawn by peripheral venipuncture into an EDTA tube and processed to obtain plasma as previously described [26]. Samples were tested as duplicate. Detection of cytokine levels was performed using MILLIPLEX® MAP Human Cytokine/Chemokine/Growth Factor Panel A (HCYTA-60K, Merck Millipore), following the manufacturer’s instructions.

### Statistical data analysis

All analyses were carried out using the R software version 4.0.1 [27]. Continuous variables were described using mean and standard deviation if normality assumptions hold true, median and interquartile range was used otherwise. Qualitative variables are presented in frequencies and percentages. The effect of IIL-FL, IIL-FH, IIH-FL and IIH-FH in transcriptomic data was studied using Kruskal–Wallis test and *p* value was adjusted for multiple comparisons using the false discovery rate proposed by Benjamini and Hochberg [28].

To explore association between tumour immune microenvironment and PFS across platinum-exposed patients, LASSO-Cox regression was carried out using glmnet package [29]. Tenfold cross-validation was used to estimate lambda value with minimum mean cross-validated error. Selected variables were then dichotomised into “High” and “Low” using maximally selected rank statistics [30] from the *maxstat* package [31].

The association of our signature with response to immunotherapy was calculated using Fisher’s exact test and model comparison between PD-L1 alone and PD-L1 plus our signature was evaluated using Chi-square approximation. *p* Values were considered significant if *p* < 0.05 based on two-sided testing.

## Results

### Identification of two principal immune transcriptomic and IHC profiles in advanced GEA

**Fig. 1:**
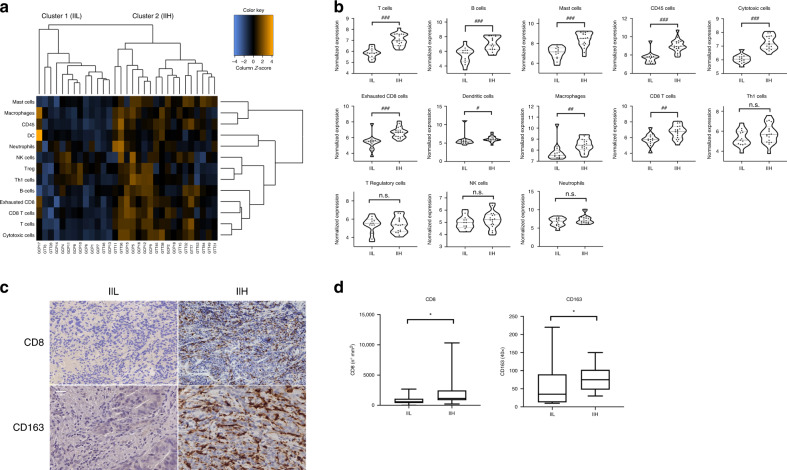
Immune transcriptomic and IHC profiles in advanced GEA. **a** Unsupervised hierarchical clustering heatmap of NanoString cell-type annotation data identifies two large clusters: the first (cluster 1), Immune infiltrate low (IIL); and the second (cluster 2), Immune infiltrate high (IIH). **b** Cytotoxic cells, CD8+ T cells, B cells, CD45+ cells, T cells, mast cells, macrophages, exhausted CD8+ T cells, Th1 cells, NK cells, dendritic cells, regulatory T cells, and neutrophils were studied. The IIH group presented with a higher representation of the immune cell population. **c** Immunostaining of CD8−/CD8+ and CD163−/CD163+ GEA in the IIL and IIH groups. Scale bar: 10 μm. **d** Statistically significant correlation between transcriptomic subgroups and immunohistochemistry of CD8 and CD163. Representation of *p* value = *<0.05; adjusted *p* value: ^#^<0.05; ^##^<0.01; ^###^<0.001.

To evaluate GEA microenvironment features, we performed a transcriptomic analysis of the primary tumour of 31 consecutive retrospective patients. Clinicopathological characteristics are provided in Supplementary Table 1. First, an unsupervised hierarchical clustering heatmap considering tumour immune microenvironment features defined by both the immune infiltrate and its immune cells subsets led us to identify two different immune subtypes of GEA: the first, characterised by a low immune infiltrate (IIL, low immune infiltrate) and the second, with a high immune infiltrate (IIH, high immune infiltrate) (Fig. 1a). A specific population analysis of several immune cells detected by the transcriptomic panel was then performed. As expected, the IIH group showed a higher representation of the immune cells (*p* < 0.05) (Fig. 1b). To validate our findings, we carried out IHC staining of all the primary tumour samples using a customised in house immune panel to evaluate the presence of CD3+, CD8+, CD163+, FOXP3+ and PD-L1+ cells. The IHC confirmed an increase in the immune infiltrate in those tumours belonging to the transcriptomic IIH subgroup. We observed that the IHC positivity for lymphocytes CD8+ (*p* = 0.03) and macrophages CD163+ (*p* = 0.03) was associated with the IIH subgroup (Fig. 1c, d). The relevance of CD8+ and CD163+ cells independently from the other markers in determining the IIH group was also remarked by our transcriptomic results (Fig. 1b). In contrast, no statistical differences were found in IIH versus IIL groups when CD3 and FOXP3 were studied by IHC (data not shown), which is also in line with what we saw at the gene expression level (Fig. 1b).

### Immune functional differences across GEA transcriptomic subtypes

**Fig. 2:**
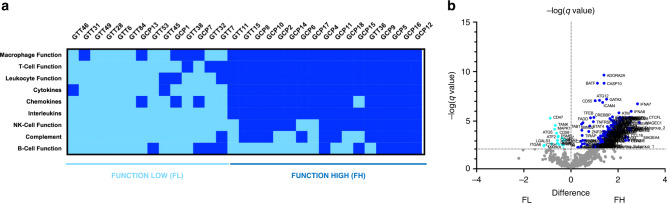
Transcriptomic functional analysis of GEA immune microenvironment. **a** GEA patient classification by immune infiltrate function (Function high, FH and Function Low, FL) according to the results of an unsupervised hierarchical clustering heatmap for each gene signature: macrophage functions, T cell function, leucocyte function, cytokines, chemokines, interleukins, NK cell function, complement and B cell function. **b** Volcano plot showing differential gene expression (FDR *q* value <0.01 (1%)) between the FH and FL subgroups.

As tumour microenvironment represents a dynamic and complex system, beyond identifying two groups based on the amount of immune infiltrate (IIL and IIH), our patients were also classified by the immune function of the cells composing the immune microenvironment. We performed an unsupervised hierarchical clustering heatmap to study T, NK, macrophages, leucocytes, and B cell functions, as well as functional signatures for cytokines, complement, chemokines, and interleukins, which clustered GEA tumours into a high or low function group for every single signature (Supplementary Fig. 2). When all the functional signatures were integrated, our cohort could be divided into two major groups: one presenting high function of cells belonging to the tumour infiltrate (*high function*, FH) and another characterised by the lack of these functions (*low function*, FL). (Fig. 2a). To better characterise these two groups, a differential gene expression analysis with a Volcano plot was performed (Fig. 2b), showing that FL tumours expressed the most proliferative genes, such as MAPK and E3 ubiquitin ligase (Supplementary Table 2), which suggested a more aggressive tumoural phenotype. As expected, low expression of immune response-related genes was found within this subgroup. On the other hand, FH tumours were enriched in immune-related genes pathways, mostly suggesting a pro-inflammatory profile. These tumours showed hyper-expression of genes related to immune cell recruitment, adaptive and innate immunity, antigen presentation, immune suppression, and inflammation (Supplementary Table 2).

### Infiltrate and functional transcriptomic integration to identify four distinct tumour immune microenvironment profiles

**Fig. 3:**
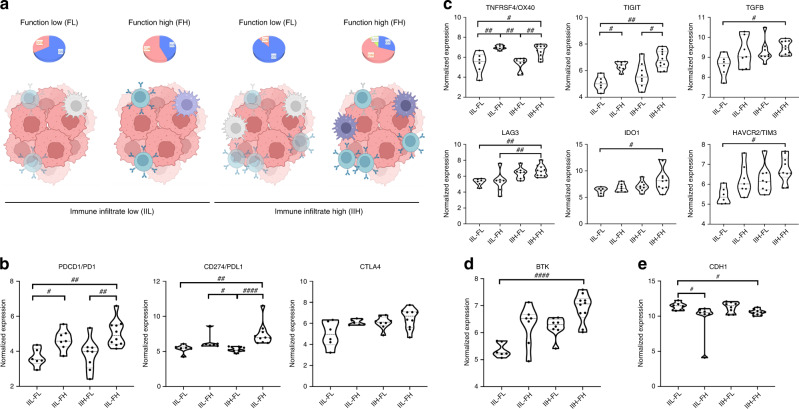
Identification of four groups according to GEA immune transcriptomic profile. **a** Classification into low and high immune infiltrate GEA. Within each group it was possible to identify the tumours with low and high immunological function. **b** Expression of the major checkpoints PD1, PD-L1, CTLA4 across the four different groups: PD1 was highly expressed in IIH-FH versus IIL-FL and in IIH-FH versus IIH-FL; PDL1 was highly expressed in IIH-FH versus IIH-FL and in IIH-FH versus IIL-FL. No differences in CTLA4 expression across the four subgroups was seen. **c** The expression of HAVCR2, IDO-1, LAG3, TIGIT, TNFRSF4, and TGFB was found to be higher in the IIH-FH versus IIL-FL subgroup. **d** Bruton’s tyrosine kinase (BTK) was highly expressed among the IIH-FH versus IIL-FL subgroup. **e** CDH1 (E-Cadherin) shows reduced expression among the IIH-FH versus IIL-FL subgroup. Representation of adjusted *p* value: ^#^<0.05; ^##^<0.01; ^####^<0.0001.

By using integrative bioinformatics assessment of both the tumour infiltrate (IIL, IIH) and the function of its component cells (FL, FH), we were able to identify 4 different tumour immune microenvironment profiles in our cohort, namely, IIL-FL, IIL-FH, IIH-FL and IIH-FH (Fig. 3a). We observed that CIN tumours mostly belonged to groups with FH while the GS subgroup presented with FL. As expected, MSI-H patients had a IIH and FH profile (Fig. 3a). Next, studying gene expression of the major check-point molecules (*PD1*, *PD-L1*, *CTLA4*) across the identified 4 subgroups, we found that PD1 and PDL1 were significantly more expressed in IIH-FH tumours than in those defined as IIL-FL, while no differences in CTLA-4 were detectable (Fig. 3b). In addition, we studied a list of relevant immunomodulatory genes such as *HAVCR2, IDO-1, LAG3, TIGIT* and *TNFRSF4*, observing that they were again overexpressed in the IIH-FH subgroup (Fig. 3c). Intriguingly, another target commonly associated with immune cells, Bruton’s tyrosine kinase (*BTK*), which is associated with lymphocyte activation, was highly expressed among the IIH-FH group (Fig. 3d). These results suggest that patients belonging to the IIH-FH group could potentially benefit from treatment with a CPI. In addition, several studies have focused on the relationship between epithelial–mesenchymal transition (EMT) and tumour microenvironment features [32, 33], prompting us to explore the correlation of the EMT with immune infiltrate and function in our cohort. In this context, IIH-FH tumours presented reduced *CDH1* expression, suggesting an involvement of the EMT in determining this phenotype (Fig. 3e). Moreover, expression of *TGF*-*β*, which also plays an important role in the EMT process [34], was higher in the IIH-FH group, supporting this hypothesis (Fig. 3c).

### Tumour immune microenvironment influence on platinum-based chemotherapy resistance

We also studied the potential role of the immune microenvironment in determining chemotherapy response. As platinum-based chemotherapy represents the gold standard for these patients, we analysed the PFS of all our patients treated with this therapy (81%). Log-rank test stratifying the population by immune infiltrate and immune function showed no differences in survival curves between the four groups (Supplementary Fig. 3A). To explore the potential relationship between any tumour immune microenvironment component and PFS across platinum-exposed patients, we performed a multivariable LASSO–Cox analysis with tenfold cross-validation, yielding a lambda value of 0.33. Variables were then dichotomised using maximally selected rank statistics. We observed that *HLA-DQA1* expression was related to worse prognosis (*p* = 0.00059) (Supplementary Fig. 3B). Conversely, high expression of *DUSP4*, *IRF4*, and *CCRL2* was associated with better response to platinum-based CT (Supplementary Fig. 3C).

### Validation of nCounter results across a prospectively collected cohort combining RNAseq and plasma cytokine profile

**Fig. 4:**
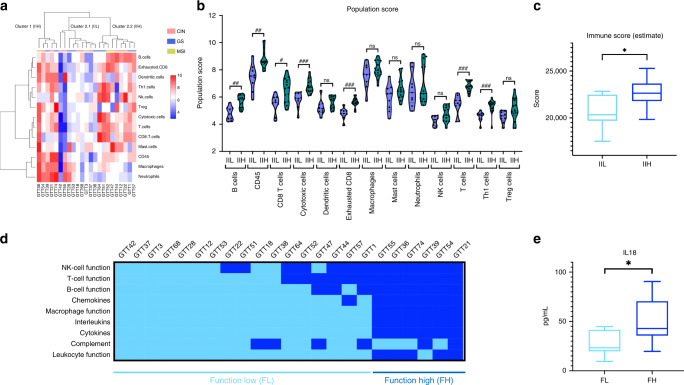
RNA analysis to study immune infiltrate features across a prospective cohort of GEA. **a** Unsupervised hierarchical clustering heatmap of the prospective-RNAseq cohort according to NanoString cell-type annotation data identifies two groups: immune infiltrate high (IIH, cluster 1 and cluster 2.2) and immune infiltrate low (IIL, cluster 2.1). **b** Cytotoxic, CD8+ T, B, CD45+, T, mast, macrophages, exhausted CD8+ T, Th1, NK, dendritic, regulatory T cells and neutrophils were studied. The IIH group had a higher representation of the immune-cell population. **c** ESTIMATE immune score showed differences between IIH and IIL groups. **d** GEA patient classification by immune infiltrate function (Function High, FH and Function Low, FL) according to the results of an unsupervised hierarchical clustering heatmap for each gene signature: NK cell function, T cell function, B cell function, chemokines, macrophage function, interleukins, cytokines, complement, and leucocyte function. **e** Plasma IL-18 levels across FL and FH groups. Representation of *p* value: *<0.05; adjusted *p* value: ^#^<0.05; ^##^<0.01; ^###^<0.001.

To validate the nCounter results, we studied immune microenvironment features of 23 consecutive patients belonging to another GEA cohort, using wider RNA sequencing analysis to overcome the limitations of the predefined panel. Clinicopathological characteristics are provided in Supplementary Table 3. The gene expression profile of the immune cells present in the tumour infiltrate dichotomises our patients into two groups, IIH (cluster 1 and cluster 2.2) and IIL (cluster 2.1) (Fig. 4a), as previously obtained with the nCounter analysis. As expected, evaluation of the immune cell scores underlined that the IIH group, has the highest total immune infiltrate (Fig. 4b). To complete the analysis, we next used a computational algorithm (the immune score of ESTIMATE) [24] for each tumour sample. Finally, it was confirmed that IIH tumours presented a high immune population (*p* = 0.028) (Fig. 4c). Furthermore, to overcome the limitation of a descriptive-only evaluation of cellular infiltrate, we added assessment of immune cell function to our study, and were subsequently able to classify our cohort into FH and FL groups (Fig. 4d). Interestingly, patients belonged to different clusters within the IIH group, suggesting a different functional profile between them. Patients belonging to Cluster 1 (Fig. 4a) were FH while FL tumours belonged to Cluster 2.2 (Fig. 4a). These results support our hypothesis that cellular function has an importance beyond infiltrate component evaluation. In this context, to explore whether tumour microenvironment function could be related to cytokine plasma levels, we also studied the plasma samples of our prospective cohort with a multi-cytokine panel. We found that patients belonging to the FH group presented higher levels of IL-18 (*p* = 0.042), a pro-inflammatory cytokine involved in Th1 response as well as in inducing IFN-γ production (Fig. 4e). This result suggests that IL-18 plasma analysis could be useful for monitoring the systemic immune response. No differences in other cytokines were detectable in our cohort.

Finally, considering the immune infiltrate and its function, we were able to classify our patients into the same four groups as previously described in our retrospective cohort. However, due to the limited sample size, only one patient belonged to the IIL-FH, thus limiting the value of the results in this group.

### IIH-FH immune microenvironment profile and immunotherapy benefit

**Fig. 5:**
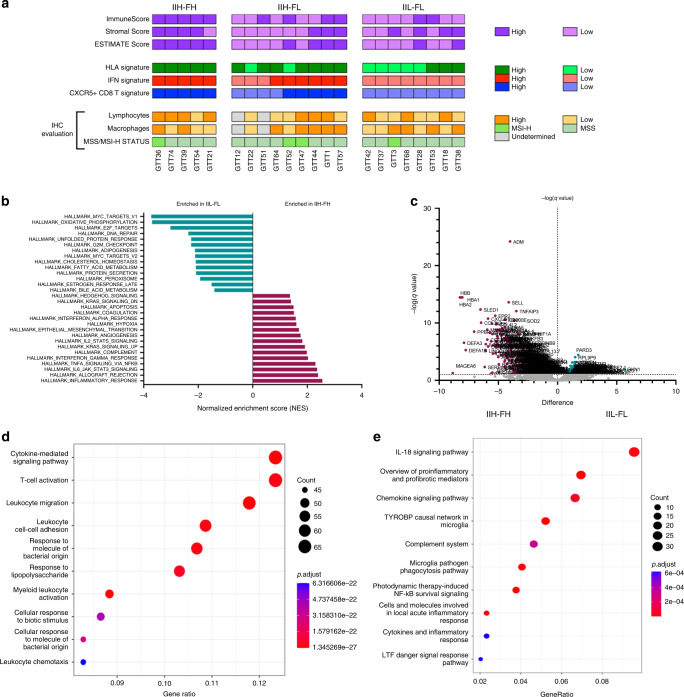
IIH-FH profile could predict response to CPIs. **a** Second cohort immune microenvironment description based on combined RNA sequencing and IHC analyses. **b** Gene Set Enrichment Analysis of IIH-FH and IIL-FL groups according to FDR < 5% (*q*-value: <0.05). Green: pathways enriched in IIL-FL. Burgundy: pathways enriched in the IIH-FH. **c** Volcano plot showing differential gene expression analysis between IIH-FH and IIL-FL groups. **d** Gene Ontology biological process enrichment analysis of the 626 differentially expressed genes recognised between IIH-FH and IIL-FL groups. **e** Functional enrichment analysis (WikiPathways) of the 626 differentially expressed genes recognised between the IIH-FH and IIL-FL groups.

As previously proposed in our nCounter cohort, patients belonging to the IIH-FH group could potentially benefit from immunotherapy. Using our second cohort as validation, RNA-seq technology was performed to evaluate immune gene sets that have been associated with CPIs response, such as the HLA and IFN signatures, and the gene expression of the major checkpoint molecules, overcoming the limitations of the nCounter panel. As expected, all the previously mentioned signatures were highly expressed in the IIH-FH group (Fig. 5a and Supplementary Fig. 4A–C). Immunomodulatory genes and *BTK* expression was also studied, and it was possible to confirm their higher expression in the IIH-FH versus the IIL-FL group (Supplementary Fig. 4D, E). A further analysis of the CXCR5+CD8+ T signature, which has been proposed as a possible biomarker of immunotherapy response in gastric cancer [35], showed high expression in IIH-FH tumours, while tumours belonging to the IIL-FL presented reduced expression (Fig. 5a and Supplementary Fig. 4F).

To analyse further differences between the IIH-FH and IIL-FL groups, we studied the 50-hallmark gene set signature through the GSEA (Gene Set Enrichment Analysis) [36]. Across the IIH-FH phenotype, we found 30 upregulated gene sets, 18 of them with an FDR < 25% (*q*-value: <0.25) (Supplementary Table 4). Among them, the inflammatory response signature, the IFN-γ and IFN-α responses, and IL-2-STAT5 signalling pathway were significantly enriched (Fig. 5b). Notably, the EMT signature was newly highly represented among the IIH-FH subgroup, highlighting the potential relation between microenvironment and tumour mesenchymal phenotype. Conversely, in the IIL-FL group we found 20 upregulated gene sets, 18 of which had an FDR < 25% (*q*-value: <0.25) (Supplementary Table 5). Among them, the E2F, MYC and G2M checkpoint targets signatures were enriched, highlighting a more proliferative phenotype (Fig. 5b). To understand the differences between IIH-FH and IIL-FL tumours we analysed the differential gene expression profile between these two groups. 626 genes were found to be differentially expressed between them (adjusted *p* value <0.05; |log2FC| > 1; Fig. 5c). A Gene Ontology biological process enrichment analysis showed that Cytokine-mediated signalling pathways, T cell activation and leucocyte migration were differentially expressed among the two profiles (Fig. 5d), suggesting a potentially key role for the immune phenotype in determining which group a tumour belongs to. Interestingly, the chemokine and IL-18 signalling pathways were significantly enriched in a functional enrichment analysis (Fig. 5e). This result suggests that IL-18 may contribute to differential functional states of the tumour immune microenvironment.

### Immune transcriptomic profile for prediction of immunotherapy response beyond CPS in a CPI-exposed cohort of advanced GEA

Our previous analyses support a role for transcriptomics in potentially identifying CPI-sensitive tumours. To further evaluate the predictive value of our immune classification, we tested it by in silico analysis in an external CPI-exposed cohort [14]. Among the CPS < 5 population, only 9.7% achieved a response from pembrolizumab (anti-PD1), while most patients with CPS ≥ 5 benefitted from immunotherapy, underlining the importance of additional biomarkers in the population with CPS < 5. When we tested our transcriptomic classification in this external cohort, we identified three immune microenvironment profiles: IIL-FL, IIH-FL and IIH-FH. In particular, 67% of immunotherapy responders belonged to the IIH-FH group. Furthermore, our classification was significantly associated with response to immunotherapy (*p* = 0.003). A logistic regression model combining CPS and our immune transcriptomic classification increased the model fit compared to CPS alone (*p* = 0.043). Beyond PD-L1 expression, therefore, our model was able to predict response in patients with CPS < 5.

## Discussion

Our study describes four distinct immune microenvironment profiles according to immune infiltrate and its function (IIL-FL, IIL-FH, IIH-FL and IIH-FH), using multifactorial assessment of transcriptomic and pathological features in patients with advanced GEA. This subclassification is of interest to potentially detect sensitivity or resistance to CPIs. Intriguingly, tumours belonging to the IIH-FH subgroup expressed the most gene signatures related to immunomodulatory pathways and immunotherapy response. These results were confirmed with an internal second cohort and finally validated “in silico” in an external CPI-exposed population, where 67% of immunotherapy responders belonged to the IIH-FH group. Furthermore, our signature was associated with response to immunotherapy (*p* = 0.003) and a logistic regression model combining CPS and our transcriptomic analyses showed that using both tools allowed more precise detection of patients who could benefit from immunotherapy than using CPS alone (*p* = 0.043).

Transcriptomic analyses are improving our knowledge of immune tumour microenvironment [37–39]. Most research has focused on studying immune infiltrate composition to understand resistance to CPIs [12, 40, 41], while there is growing evidence that the functional status of the immune microenvironment marks the difference in immunotherapy response [42–44]. Analysis of immune function could therefore help in determining sensitivity or resistance to immunotherapy. In this regard, our gene expression analysis showed that FH tumours were mostly enriched in immune-related genes, while FL tumours principally expressed genes involved in cell proliferation. Further, by examining both the infiltrate composition and its function we were able to describe distinct immune microenvironment profiles: IIL-FL, IIL-FH, IIH-FL and IIH-FH. Of interest, it was confirmed that tumours belonging to the IIH-FH subgroup expressed the most immunomodulatory genes, such as *TIM3, IDO-1, LAG3, TIGIT* and *OX40*, making them potential candidates for classic CPI (anti PD-L1, PD1, CTLA4) or novel immunotherapy combinations such as anti-LAG3 and anti-TIM3 bispecific antibodies which could overcome primary or secondary resistance [45]. To complete our study, we also evaluated intrinsic tumour features, such as EMT. The bilateral dynamical interaction between EMT and microenvironment in determining a pro- or anti-inflammatory profile has been recently described in several tumour models [46]. Our results showed that IIH-FH tumours presented reduced expression of *CDH1* and higher expression of *TGF*-β, suggesting a mesenchymal phenotype.

The prognostic role of tumour microenvironment in predicting response to chemotherapy has been investigated in several studies [47, 48]. In our cohort, the aim was to assess whether belonging to any of the four previously identified immune groups could have a prognostic role with respect to PFS with first line platinum-based chemotherapy. Cox regression analysis showed no differences between the 4 groups, although with LASSO-Cox analysis HLA-DQA1 expression was related to worse prognosis (*p* = 0.00059). The association between this HLA and platinum resistance has already been observed in other relevant research, even at a single cell level [49].

The NCounter allowed us to identify the four previously mentioned immune microenvironment profiles, based on population and functional analysis. To validate this immune classification and overcome the limitation of the number of genes included in an array panel, we used RNA sequencing to analyse the transcriptome of a prospective cohort of advanced GEA patients, who shared the same clinic and pathological characteristics as the first retrospective cohort. In this case, we were able to divide the population into the same four subgroups. As proposed, the function of those cells composing the tumour immune microenvironment could play a key role in determining sensitivity or resistance to CPIs. In this regard, we found that patients belonging to the FH group presented higher plasma levels of IL-18 (*p* = 0.042) than those defined as FL. IL-18 is a pro-inflammatory cytokine involved in Th1 response as well as in inducing IFN-γ production [50]. This result suggests that regardless of immune infiltrate, FH tumours may exhibit high antitumoural activity and a systemic pro-inflammatory profile. Furthermore, differential gene expression analysis showed that IL-18 signalling pathway was significantly more enriched in IIH-FH tumours than IIL-FL ones. The role of IL-18 in relation to immune activity and immunotherapy warrants further studies in a larger cohort of GEA patients. As expected, IIH-FH exhibits EMT and an inflamed phenotype, with high expression of genes related to HLAs, interferon-γ activation and immune checkpoints. In particular, the inflammatory response signature, IFN-γ and IFN-α responses and the IL-2-STAT5 signalling pathways were significantly enriched among the IIH-FH subgroup, as shown in GSEA analysis. These results confirmed the previous ones observed in the retrospective nCounter cohort.

Finally, to evaluate whether this transcriptomic classification could help identify patients who would potentially benefit from CPI, we studied a cohort of GEA patients treated with pembrolizumab as second or third line. Interestingly, 67% (8/12) of immunotherapy responders belonged to the IIH-FH group. Across this cohort, our signature was associated with response to immunotherapy (*p* = 0.003). Moreover, the logistic regression model combining CPS and our transcriptomic analysis showed that use of both tools increased the ability to detect patients who could benefit from immunotherapy versus CPS alone (*p* = 0.043). An important finding in our study is that all patients belonging to the first and second cohorts had CPS < 5, potentially indicating a marginal benefit of adding immunotherapy. However, CPI sensitivity was found when an active, highly functional immune microenvironment was confirmed in our transcriptomic analysis.

This study presents some limitations derived from its exploratory design, such as the small sample size for both retrospective and prospective cohorts without previous exposure to immunotherapy, and the validation of our results performed in silico in a public cohort.

In conclusion, our work allows the identification of an immune-enriched subtype of advanced GEA, characterised by high immune cell activation, which could potentially help achieve a more precise immunotherapy approach beyond CPS. Further evaluation of the capabilities of this transcriptomic classification are needed in wider cohorts of CPI-exposed patients.

## Supplementary information

Supplementary tables and figures with legends

## Data Availability

All data relevant to the study are included in the article or uploaded as supplementary material. The data sets redacted, the statistical analysis plan and individual participants’ data supporting the results reported in this article will be made available from the corresponding author on reasonable request.
