# Supplementary material for: Integrative immune transcriptomic classification improves patient selection for precision immunotherapy in advanced gastro-oesophageal adenocarcinoma
Source: Br J Cancer. 2022 Oct 17;127(12):2198–206. doi: 10.1038/s41416-022-02005-z (PMC9727124; doi:10.1038/s41416-022-02005-z)
Supplement: Supplementary file 1 — Supplementary tables and figures with legends [file 41416_2022_2005_MOESM1_ESM.docx]

**SUPPLEMENTARY INFORMATION**

**Supplementary Table 1. Baseline patient characteristics. Retrospective-nCounter cohort (N=31)**

| **Median age (range)** | **67 (42-87)** |
| --- | --- |
| **SEX** | |
| Male | 19 (61) |
| Female | 12 (39) |
| **Lauren subtype (%)** | |
| Diffuse | 12 (39) |
| Intestinal | 15 (48) |
| Mixed | 4 (13) |
| **Her2 status (%)** | |
| IHC 2+ FISH positive or 3+ | 16 (51) |
| IHC 0-1-2 FISH negative | 8 (26) |
| Undetermined | 7 (23) |
| **MSS status(%)** | |
| MSS | 30 (97) |
| MSI | 1 (3) |
| **cps (%)** |  |
| <1 | 18 (59) |
| 1-5 | 11 (35) |
| NA | 2 (6) |
| **Tumor location** | |
| Cardias | 6 (19) |
| Body-Fundus | 9 (29) |
| Antrum | 16 (52) |
| **Clinical stage** | |
| Stage III | 3 (10) |
| Stage IV | 28 (90) |
| **Therapeutic approach at diagnosis** | |
| Peri-operative platinum-based chemotherapy | 3 (10) |
| Platinum-based chemotherapy 1L | 22 (71) |
| Pembrolizumab | 1 (3) |
| BSC | 5 (16) |

**Supplementary Table 2. Differential Gene expression analysis between FH and FL groups**


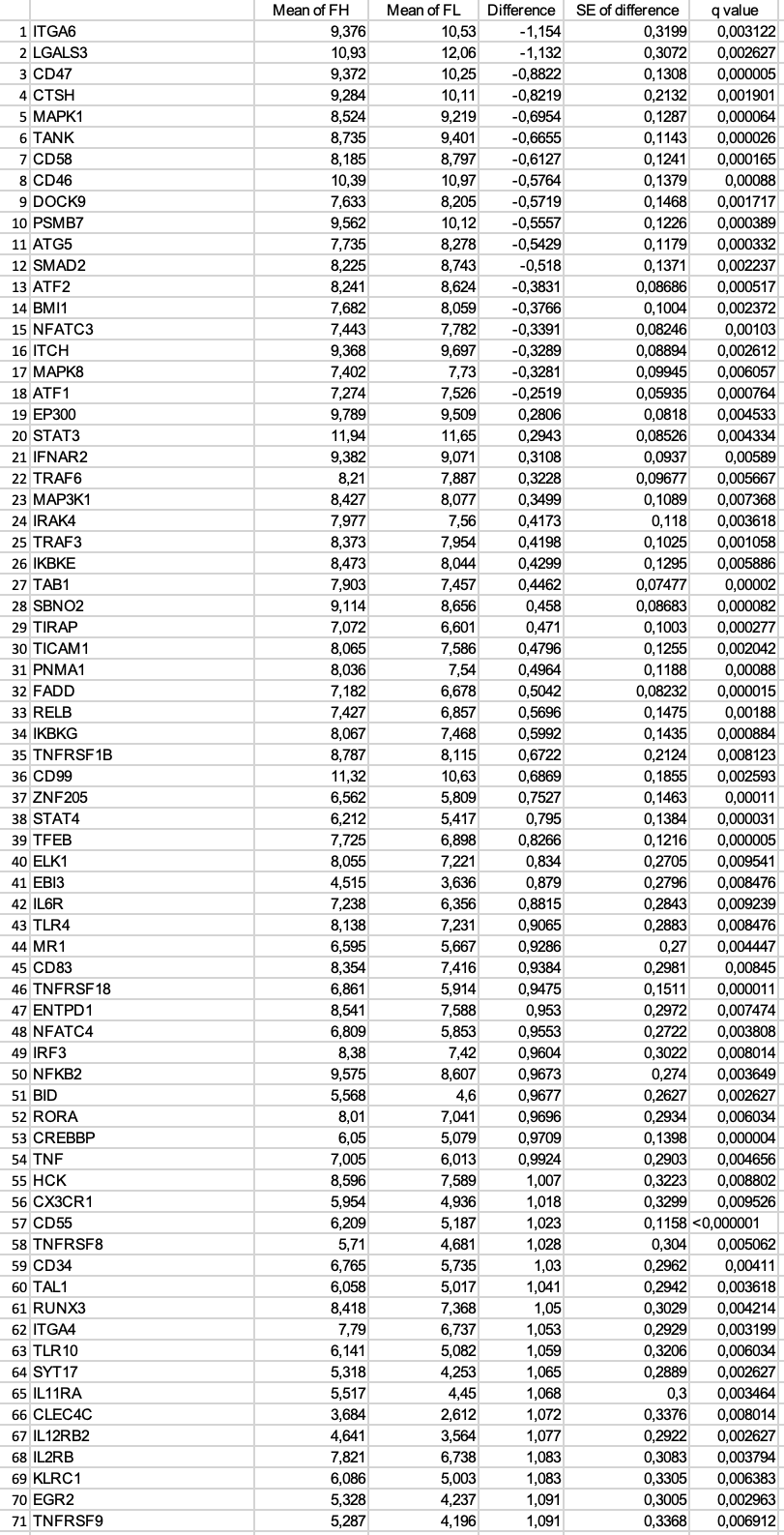


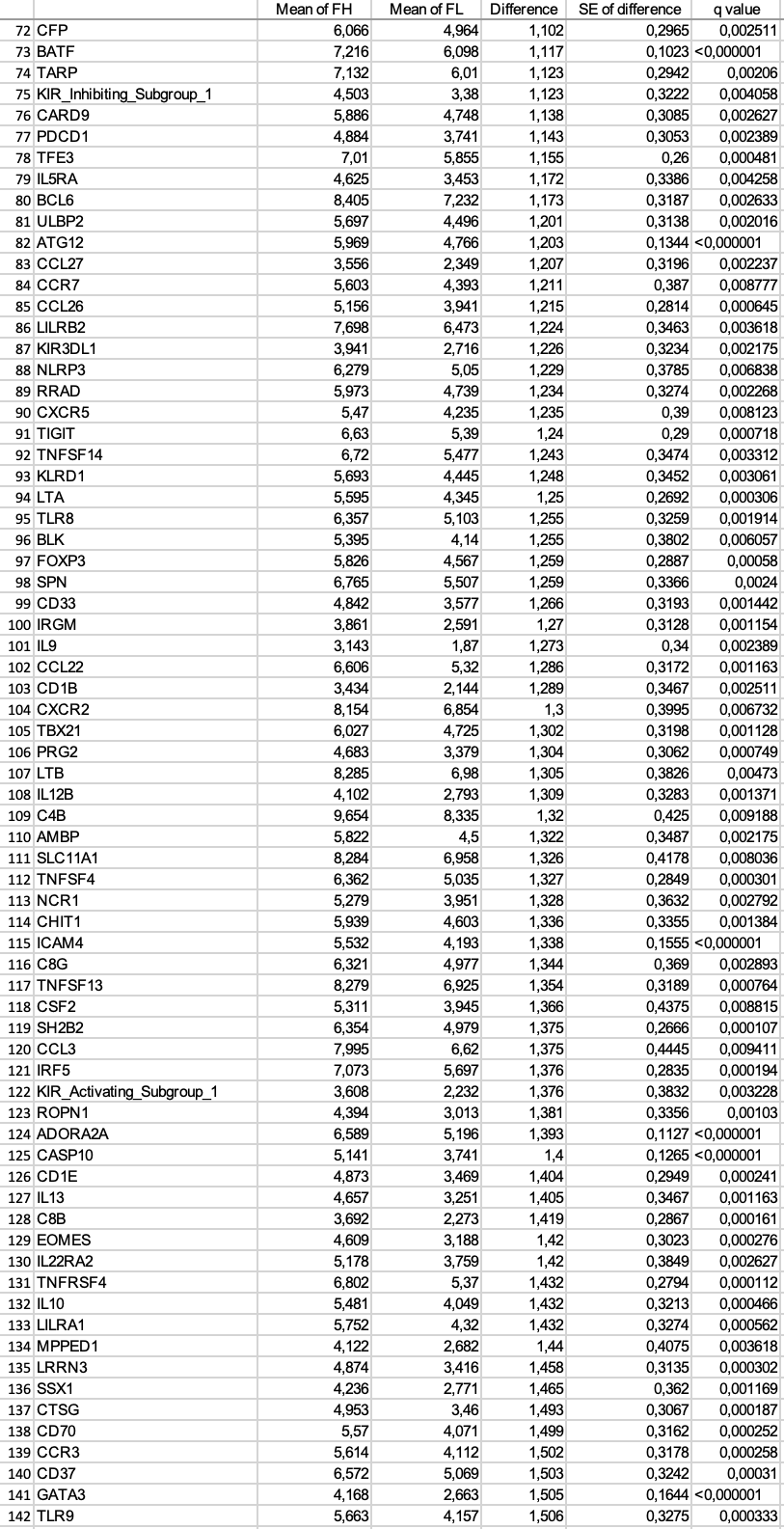


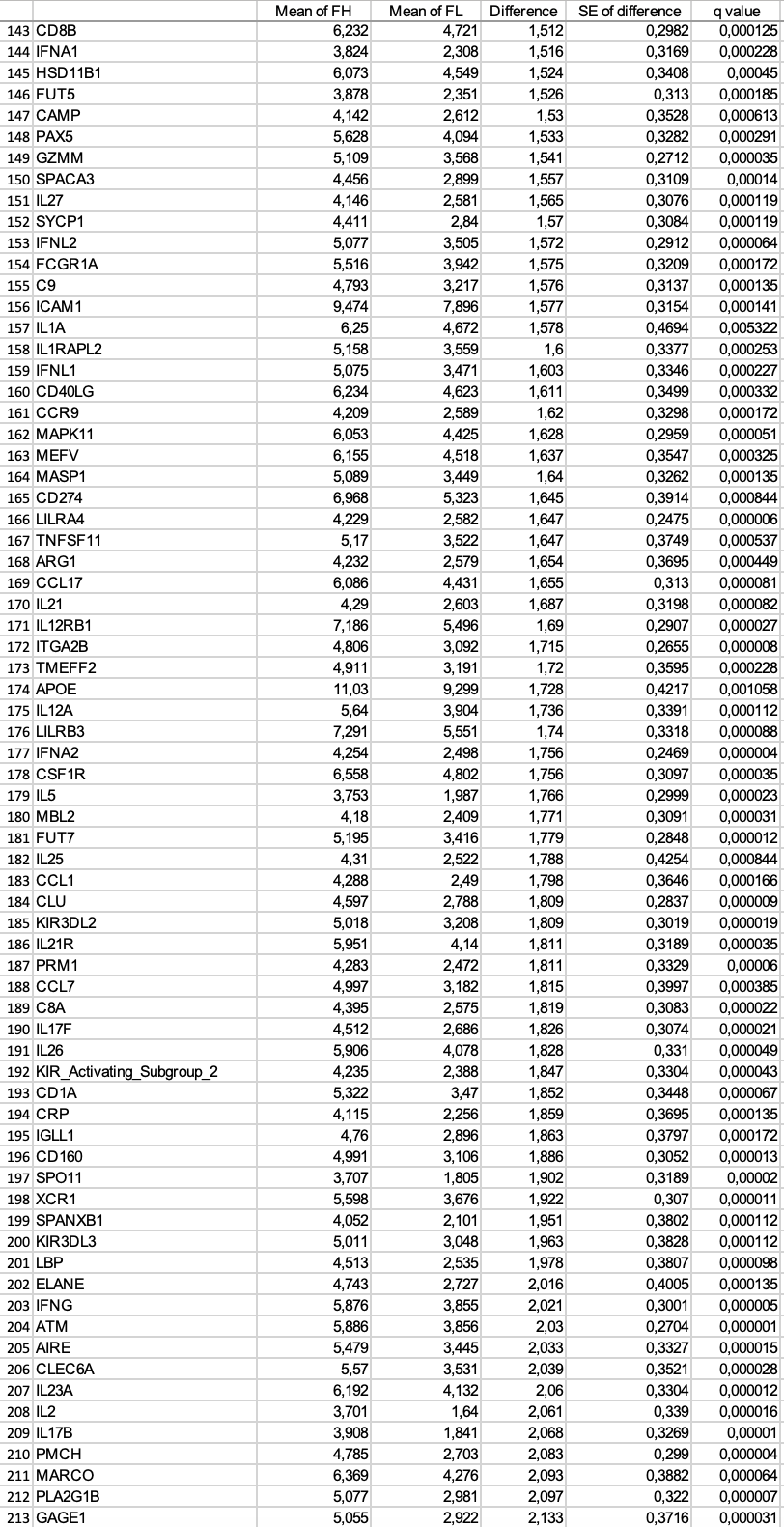


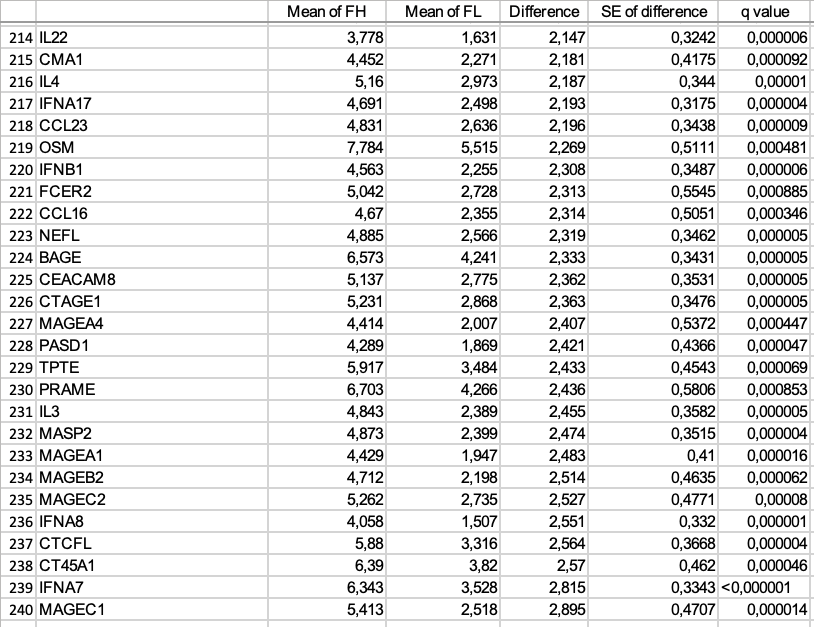


**Supplementary Table 3. Baseline patient characteristics. Prospective-RNAseq cohort (N=23)**

| **Median age (range)** | 73 (54-91) |
| --- | --- |
| **sex** | |
| Male | 17 (74) |
| Female | 6 (26) |
| **Lauren subtype (%)** | |
| Diffuse | 6 (26) |
| Intestinal | 13 (57) |
| Mixed | 4 (17) |
| **Her 2 status (%)** | |
| IHC 2+ FISH positive or 3+ | 3 (13) |
| IHC 0-1-2 FISH negative | 20 (87) |
| Undetermined |  |
| **MSS status(%)** | |
| MSS | 19 (83) |
| MSI | 4 (17) |
| **CPS (%)** |  |
| <1 | 12 (52) |
| 1-5 | 8 (35) |
| NA | 3 (13) |
| **Tumor location** | |
| Cardias | 6 (26) |
| Body-Fundus | 6 (26) |
| Antrum | 11(48) |
| **Clinical stage** | |
| Stage III | 1 (4) |
| Stage IV | 22 (96) |
| **Therapeutic approach at diagnosis** | |
| Platinum based chemotherapy 1L | 12 (52) |
| Pembrolizumab | 3 (13) |
| BSC | 6 (26) |
| Palliative gastrectomy | 2 (9) |

**Supplementary Table 4. GSEA hallmark gene signatures upregulated in IIH-FH vs IIL-FL phenotype.**

**Supplementary Table 5. GSEA hallmark gene signatures upregulated in IIL-FL vs IIH-FH phenotype.**

**Supplementary Figure 1. CPS evaluation in GEA**


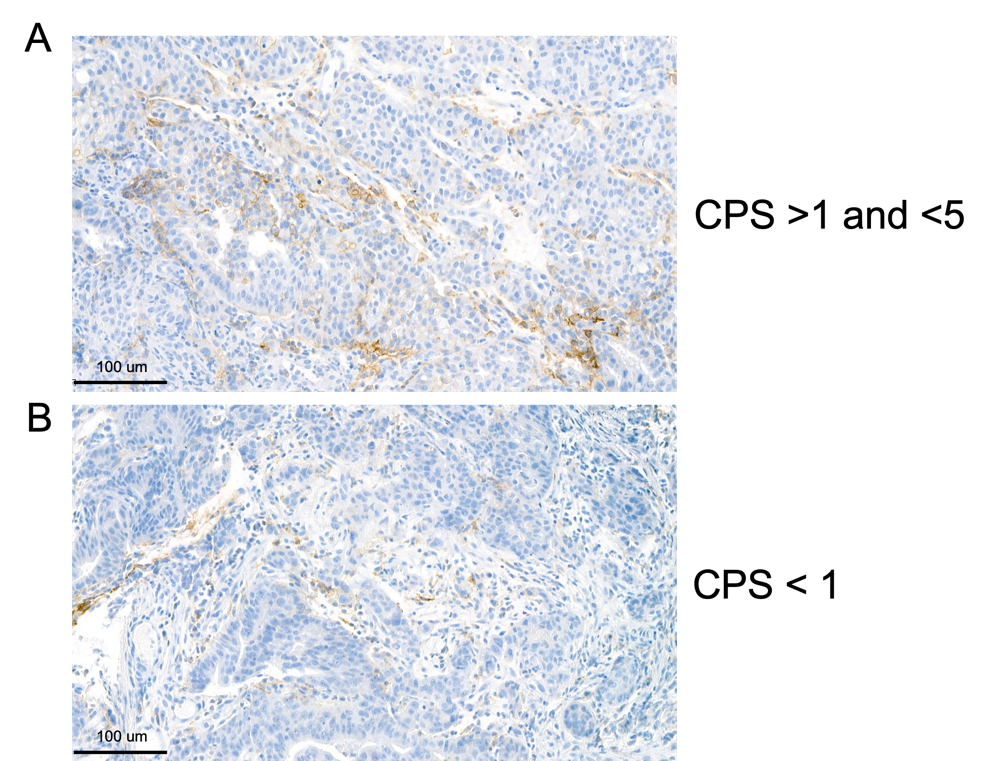


**Supplementary Figure 1**. CPS evaluation. (A) Tumor sample presenting CPS>1 and <5. (B) Tumor sample presenting CPS<1. Scale bar: 100 μm.

**Supplementary Figure 2. Unsupervised hierarchical clustering heatmaps to define immune infiltrate function**

**Supplementary Figure 2**. Unsupervised hierarchical clustering heatmap for each gene signature: T Cell Functions, Leukocyte Functions, Interleukins, Cytokines, Macrophage Functions, Complement, Chemokines, B-Cell Functions and NK-Cell Functions. The analysis allows the identification of two groups: FH and FL.

**Supplementary Figure 3. LASSO-Cox analysis to study tumor immune microenvironment influence on platinum-based chemotherapy response**

**Supplementary Figure 3.** (A) No differences in progression-free survival (PFS) were seen in the 4 immune microenvironment profiles. (B) LASSO-Cox analysis showed that high HLA-DQA1 expression was related to worse prognosis (p=0.00059). (C) LASSO-Cox showed that high expression of DUSP4, IRF4, and CCRL2 were associated with better response to platinum-based CT.

**Supplementary Figure 4. RNA sequencing analysis to evaluate several already known immune signatures and major immune modulatory pathways.**


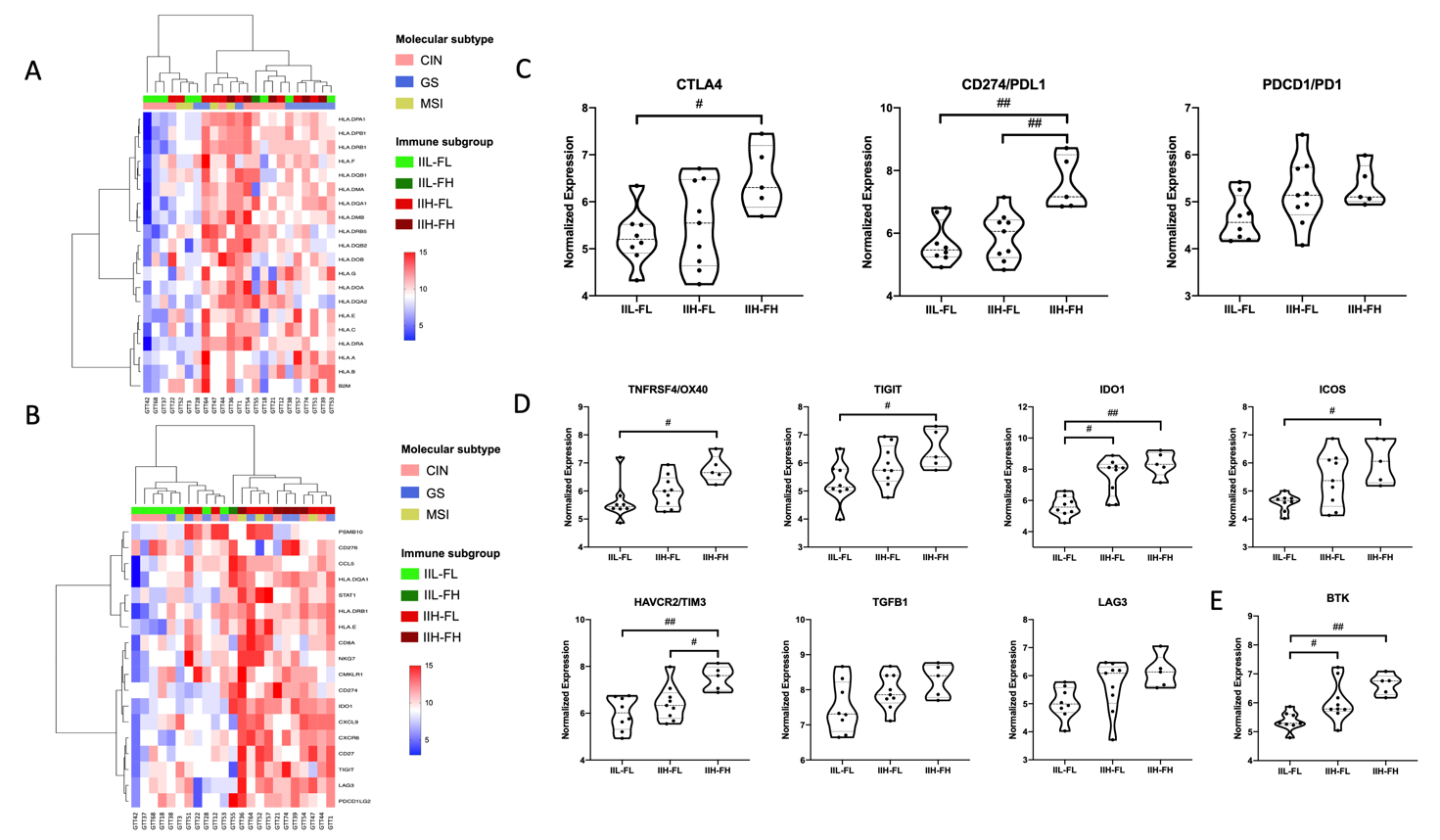


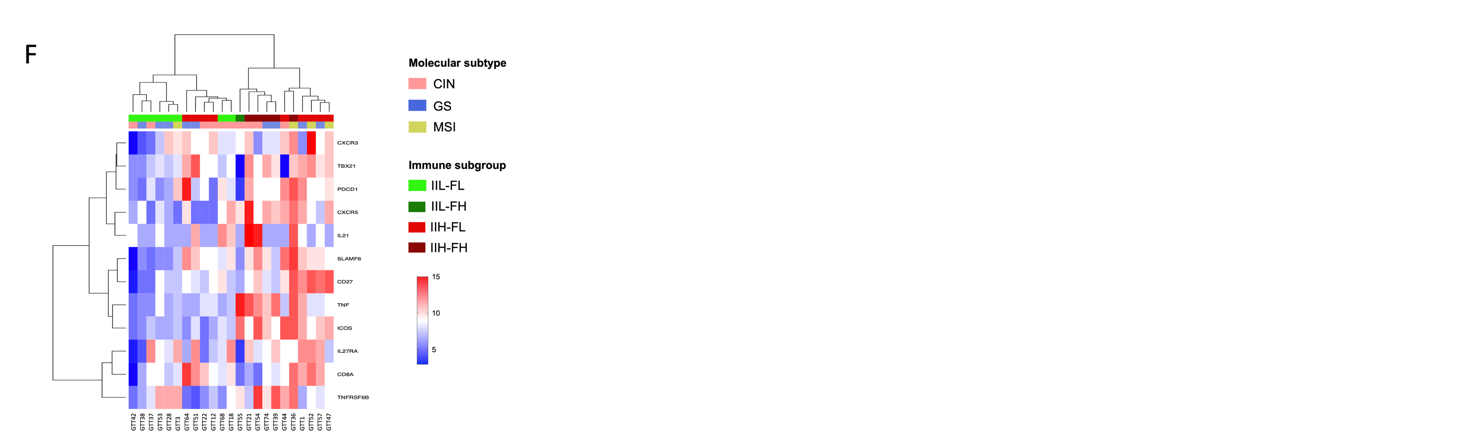


**Supplementary Figure 4**. Unsupervised hierarchical clustering heatmap of RNA sequencing to evaluate HLA (A) and IFN (B) signatures across GEA patients. (C) Expression of the major checkpoints *PD1, PD-L1, CTLA4* across the different groups: *CTLA4* was highly expressed in IIH-FH versus IIL-FL; *PD-L1* was highly expressed in IIH-FH versus IIL-FL and in IIH-FL. No differences in *PD1* expression across the subgroups were observed. (D) Expression of *TNFRSF4, ICOS, TIGIT, HAVCR2* and *IDO-1* was found to be higher in the IIH-FH versus IIL-FL subgroup. No differences in *TGFB1* and *LAG3* were seen among the groups. (E) Bruton’s tyrosine kinase (*BTK*) was highly expressed among the IIH-FH versus IIL-FL subgroup. (F) Unsupervised hierarchical clustering heatmap of RNA-sequencing to evaluate CXCR5+ CD8+ T signature across GEA patients.
